# Non-Exudative Macular Neovascularization in Various Acquired Macular Degenerations with Double- and Triple-Layer Sign on OCT

**DOI:** 10.3390/diagnostics16030497

**Published:** 2026-02-06

**Authors:** Joanna Gołębiewska, Ilona Katarzyna Jędrzejewska, Justyna Mędrzycka, Mariusz Przybyś, Radosław Różycki

**Affiliations:** 1 Department of Ophthalmology, Military Institute of Aviation Medicine, 01-755 Warsaw, Poland; 2 “Świat Oka” Ophthalmology Center, 02-511 Warsaw, Poland

**Keywords:** non-exudative macular neovascularization, double-layer sign, triple-layer sign, age-related macular neovascularization, pachychoroid, exudative MNV

## Abstract

**Background/Objectives**: To investigate the rate of exudative progression over time in patients with non-exudative macular neovascularization (NE-MNV) associated with various acquired macular degenerations presenting with a double-layer sign (DLS) or triple-layer sign (TLS) on optical coherence tomography (OCT), and to identify potential predictors of this progression. **Methods**: Fifty-one eyes of fourty-nine patients with a DLS or TLS on OCT images were identified. OCT angiography (OCTA) was performed to detect NE-MNV, and only eyes with confirmed NE-MNV were included in the final analysis. Central macular thickness (CMT), choroidal thickness (CT), morphology of the abnormal vessels, the duration of follow-up, progression to active exudative MNV, and the status of the contralateral eye were assessed. **Results**: The final analysis included 32 eyes of 30 participants with NE-MNV. The median observation period was 46 months. The causes of NE-MNV were age- related macular degeneration (AMD) in 59.38% of eyes, pachychoroid epitheliopathy (PPE) in 37.50%, and other causes in 3.12%. Exudation developed in 15.62% of eyes (median time to onset: 24 months), predominantly in the AMD subgroup. Abnormalities in the fellow eye were present in 59.38% of cases. Neither age nor other factors, including sex, cause of MNV, CMT, CT, MNV morphology, or fellow eye status, were statistically significant predictors of progression to active MNV (*p* = 0.67, *p* > 0.99, *p* = 0.62, *p* = 0.09, *p* = 0.09, *p* = 0.2, *p* = 0.62, resp.). **Conclusions**: NE-MNV is an asymptomatic condition that may occur in the course of various retinal diseases. While DLS and TLS demonstrate high sensitivity and specificity for the diagnosis of NE-MNV, their presence does not always indicate concurrent MNV. Multimodal imaging is essential for accurate monitoring of these patients and detection of potential disease progression.

## 1. Introduction

Macular neovascularization (MNV) is defined as the growth of new, abnormal blood vessels, originating from the choroidal or deep retinal circulation. Active MNV typically leads to exudation and subsequent vision decrease. In 2013, Querques et al. first described a non-exudative MNV (NE-MNV) in the course of age-related macular degeneration (AMD), introducing the terminology “treatment-naïve quiescent” MNV [1]. Since then, numerous studies have investigated the occurrence, prevalence, and natural history of these lesions. The entity has prompted the proposal of various terminologies, including ‘quiescent’, ‘subclinical,’ or ‘non-exudative’ MNV [2]. Non-exudative MNV usually refers to the entity of treatment-naïve type 1 neovascularization in the absence of associated signs of exudation: subretinal fluid (SRF), intraretinal fluid (IRF), and/or presence of subretinal hyperflective material (SHRM). This lesion may occur in the course of various conditions, including: AMD, myopic degeneration [3], pachychoroid spectrum disorders, angioid streaks (AS), and inherited chorioretinal dystrophies. The pachychoroid phenotype was first described by Warrow et al. in 2013 and is characterized by an abnormally thickened choroid, often with dilated outer choroidal vessels, associated with retinal pigment epithelium (RPE) alterations in the absence of other causes of choroidal thickening [4].

Various studies have demonstrated that MNV may remain in a non-exudative state for a variable period, and patients typically stay asymptomatic. Optical coherence tomography (OCT) provides high-resolution imaging of the retina and choroid and is crucial in detecting even small amounts of exudation. OCT angiography (OCTA) is currently the best technique for imaging and analyzing changes in retinal microcirculation associated with various ophthalmic and systemic diseases. OCTA delivers highly detailed, three-dimensional images of the entire microvasculature of the retina and choroid and helps to assess retinal perfusion or confirm MNV without intravenous dye injection. The presence of a DLS (RPE and Bruch’s membrane) or TLS (RPE, neovascular tissue, Bruch’s membrane) on structural OCT is a highly sensitive and specific factor in detecting NE-MNV, and OCTA should be performed in these patients to confirm the diagnosis [5]. Therefore, early detection of non-exudative MNV in asymptomatic patients is crucial in identifying eyes at high risk for exudation and visual decline [6,7].

The aim of our study was to investigate the rate of exudative progression over time in patients with NE-MNV in various acquired macular degenerations with double- and triple-layer sign on OCT and to attempt to identify predictors of this progression.

## 2. Material and Methods

This retrospective study included 51 eyes of 49 patients presenting with DLS and/or TLS visible in OCT images. Patients were enrolled in the Outpatient Clinic of the Military Institute of Aviation Medicine in Warsaw (Poland) between January 2021 and April 2025. The study protocol was approved by the Bioethics Committee at Lazarski University and was carried out in accordance with the tenets of the Declaration of Helsinki.

The inclusion criterion was the presence of the DLS or TLS visible on OCT images in one or both eyes, occurring in the course of various acquired macular degenerations. Exclusion criteria were: the presence of any signs of exudation (subretinal fluid, intraretinal fluid, subretinal hyperreflective material), prior anti-vascular growth factor (anti-VEGF) treatment in the study eye, and macular pathologies that could influence the results, including diabetic retinopathy, hereditary retinal dystrophies, vitreo-retinal diseases, and a history of uveitis. All patients underwent a complete ophthalmic examination, including slit lamp biomicroscopy with dilated fundus examination, best-corrected visual acuity (BCVA) assessed using the Snellen fraction, refractive error, and intraocular pressure measurement. Multimodal imaging was performed using a Swept-Source OCT (SS-OCT) device (DRI OCT Triton; Topcon, Tokyo, Japan) and included color fundus photography, fundus autofluorescence, structural OCT, and OCT angiography. A 9 mm radial OCT B-scan centered on the fovea was performed to obtain high-quality images of the retina and choroid. On OCT images, we assessed the following features: retinal structure, retinal pigment epithelium abnormalities, presence of the DLS and/or TLS, presence of SRF, IRF, and SHRM. Central macular thickness (CMT) (μm) data were obtained from retinal maps using the same device. Choroidal thickness (CT), defined as the distance between the hyper-reflective line corresponding to the base of the RPE and the hyper-reflective line corresponding to the chorioscleral interface, was measured manually. OCTA was performed using 4.5 × 4.5 mm scans centered on the macula. In the OCTA images, we assessed retinal and choroidal blood flow, focusing primarily on the presence of abnormal flow within the MNV network. The OCTA images were analyzed using a customized segmentation approach to optimize MNV visualization. The outer retina slab was defined from the outer plexiform layer (OPL) to Bruch’s membrane, while the choriocapillaris slab was defined as a 20–30 μm thick layer starting immediately below the RPE or Bruch’s membrane. All scans were manually checked to ensure correct segmentation and to identify poor-quality scans (e.g., those with motion artifacts or blurred images) where data were insufficient for proper analysis.

We also evaluated the morphology of the neovascular vessels and classified them as: glomerulus, medusa, sea fan, long linear vessels, or indistinct [8]. Eyes with low-quality scans (image quality < 60), motion artifacts or blurred images were excluded from the analysis.

## 3. Statistical Procedures

Categorical variables were depicted through integer numbers and percentages (frequencies). Numerical traits were described as their mean, median, standard deviation, and lower-to-upper quartile values. The normality of distribution was assessed by using the Shapiro–Wilk W test. Due to small group sizes, non-parametric tests were used. The Mann–Whitney U test was fitted in order to test age differences. Contingency tables with the aforementioned frequencies were accompanied by computing Fisher’s exact test. A level of *p* < 0.05 was deemed statistically significant. All the routines were carried out using Statistica™, release 13.3 (TIBCO Software Inc., Palo Alto, CA, USA).

## 4. Results

Initially, 51 eyes of 49 patients presenting with the DLS and/or TLS visible on OCT images were qualified for the study. Following OCTA confirmation of NE-MNV, 32 eyes of 30 participants (15 female, 15 male) were included in the final analysis. This indicated that MNV was found in 62.74% of these eyes. The mean age of the subjects was 64.09 years (range: 37–88 years; SD = 15.48). All participants were Caucasian. Patients’ demographic and clinical characteristics were summarized in Table 1.

**Figure 1:**
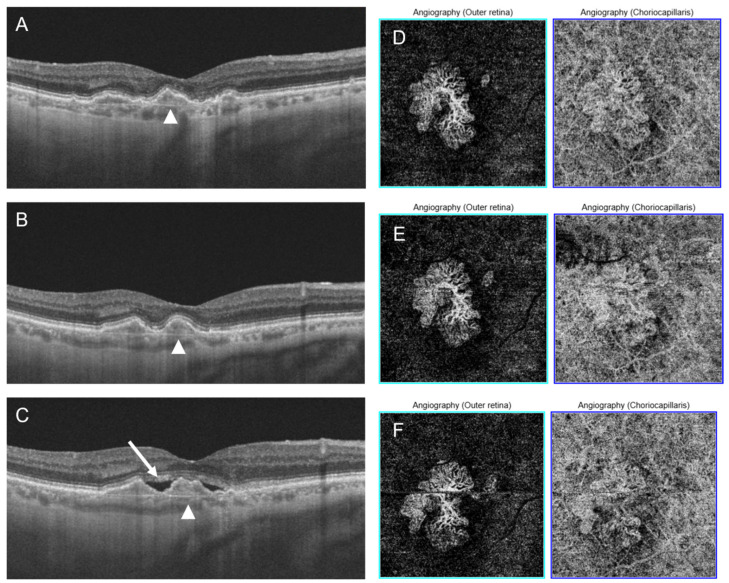
Conversion to active MNV in a patient with AMD. OCT images (**A**–**C**) show TLS (arrowheads) and subretinal fluid (**C**, arrow). (**A**) Baseline examination. (**B**) Follow-up examination at 12 months. (**C**) Follow-up examination at 18 months. OCTA images at the level of outer retina and choriocapillaris (**D**–**F**) show the MNV network.

**Figure 2:**
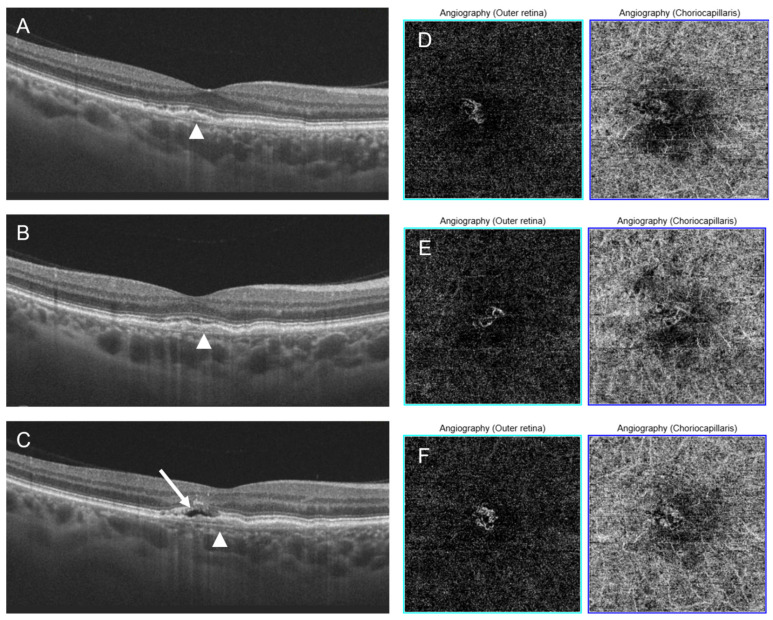
Conversion to active MNV in a patient with PPE. OCT images (**A**–**C**) show TLS (arrowheads) and subretinal fluid (**C**, arrow). (**A**) Baseline examination. (**B**) Follow-up examination at 4 months. (**C**) Follow-up examination at 6 months. OCTA images at the level of outer retina and choriocapillaris (**D**–**F**) show the MNV network.

The median observation time was 46 months (Q1 = 19 months, Q3 = 54 months). The cause of NE-MNV was AMD in 59.38% of the eyes (19 patients), pachychoroid pigment epitheliopathy (PPE) in 37.50% (12 patients), and other causes in 3.12%. During the follow-up period, subretinal fluid developed in 5 eyes (15.62%), in 4 of them in the course of AMD, in 1 as a complication of PPE (Figure 1 and Figure 2). The median time to conversion was 24 months (Q1 = 15 months, Q3 = 37 months). All active MNV were classified as type 1 MNV.

The condition of the fellow eye was normal in 40.63%, and abnormal in 59.38%. We observed different morphologies of abnormal vessels with similar frequency. Neither age nor other factors, such as gender, cause of MNV, MNV morphology, CMT, CT, and status of the fellow eye, were statistically significant predictors of progression NE-MNV to active MNV (*p* = 0.67, *p* > 0.99, *p* = 0.62, *p* = 0.2, *p* = 0.09, *p* = 0.09, *p* = 0.62, resp.), as shown in Table 2.

## 5. Discussion

NE-MNV is defined as the asymptomatic presence of treatment-naïve Type 1 neovascularization without associated signs of exudation. As it may occur in the course of various conditions, its aetiology and progression pathways are likely dependent on the underlying disorder.

Research is still evolving to better understand the natural history of NE-MNV and develop appropriate treatment regimens. Many studies highlight that biomarkers play a significant role in predicting the risk of exudation and subsequent progression from NE-MNV. There is an ongoing discussion about whether NE-MNV should be treated with anti-VEGF injections. It is generally accepted that in active neovascularization, prompt initiation of anti-VEGF therapy leads to better visual outcomes by limiting structural damage of the retina and subsequent atrophic changes. Our study analyzed 32 eyes with NE- MNV, with the majority of cases associated with AMD and pachychoroid. Currently available studies mainly consider the occurrence of NE-MNV in the setting of AMD, although increasing attention is being paid to its manifestation in pachychoroid spectrum disorders. In our cohort, conversion to exudation occurred in 15.62% of the eyes, primarily within the AMD subgroup (4 of 5 eyes). The median time to conversion was 24 months. Our findings are not consistent with other reports, which generally indicate a higher rate of progression to the exudative stage. A systematic review and meta-analysis assessing the course of NE-MNV in AMD reported that exudative progression occurred in 20.9% of eyes at 1 year and in 30.7% at 2 years. Similarly, findings from an individual participant meta-analysis showed exudative progression in 18.9% of eyes at 1 year and 31.3% at 2 years [9]. The higher rate of conversion was also confirmed in a two-year observational study involving 227 eyes with unilateral exudative AMD. The overall prevalence of NE-MNV in the fellow eyes was 13.2%. It was found that the exudation risk for eyes with subclinical MNV increased from 24% after one year of follow-up to 34.5% after 2 years of follow-up [10]. In a separate study aimed at assessing the short-term history of NE-MNV, the conversion rate from NE-MNV to the exudative stage was also higher than in our cohort. This AMD study involved 40 eyes with NE-MNV, all classified as type 1 MNV. During follow-up, 32.5% progressed to an exudative stage, with a mean time to exudation of 12.6 months [11]. These differences can be partly explained by the conclusions formulated by some authors. Querques et al. investigated different clinical and anatomical features in treatment-naïve NE-MNV secondary to AMD. Based on their observations, they identified two distinct patterns for subclinical MNV: ‘subclinical MNVs’, characterized by short-term activation, and ‘quiescent MNVs’, characterized by a low rate of growth and possible long-term activation. They examined 32 eyes and, during the follow-up period (mean duration: 22 ± 9 months), 13% showed exudation before 6-month follow-up. In total, 68% did not develop signs of exudation, and 19% developed exudation after the minimum 6-month follow-up period. Their report emphasizes that the analysis of OCTA features could predict short-term activation for subclinical MNV, but no characteristic was found to predict long-term activation [1]. It is possible that ‘quiescent MNV’ was predominant in our patients, but the group is too small to draw such a conclusion. In our analysis, the only factor that demonstrated a potential risk of progression from NE-MNV to the exudative stage was the underlying disease, but the result was not statistically significant. Among eyes that developed exudation, 80% were associated with AMD. Neither the morphology of MNV nor the condition of the fellow eye revealed any significant role. Additionally, our study did not reveal any statistically significant correlation between CMT, CT, and the presence of subretinal fluid. These results are consistent with previous studies reporting no association between mean CT and the short-term development of exudation [12]. Nevertheless, several studies indicate specific features associated with faster progression to the active stage. These characteristics, easily detectable using OCT, may serve as important biomarkers in predicting disease evolution and guiding potential treatment.

A 2024 meta-analysis pointed to several risk factors associated with fast exudative progression. The most significant of these were the presence of subretinal lipid globules, large MNV area, rapid MNV growth, growth in pigment epithelium detachment height and width, the appearance of a branching pattern, and the development of a hyporeflective halo around the MNV [9].

Based on existing research, we can conclude that OCTA allows for the qualitative and quantitative evaluation of NE-MNV in the course of various diseases, especially if it is performed regularly. Researchers have demonstrated that growing MNV is associated with higher probabilities and a faster onset of exudation compared to stable MNV. Wang et al. observed that eyes with growing MNV (defined as an increase in area ≥50% within 12 months) had an increased risk of exudation and developed exudation earlier than eyes with stable MNV [13].

Reports indicate that membrane morphology may be an important factor. In our observation, the overall prevalence of specific MNV shapes was comparable; however, sea fan patterns appeared the least frequently. Notably, among the MNVs that develop exudation, we observed only the medusa shape (60%) and long linear vessels (40%).

Karacorlu et al. evaluated morphologic patterns of choroidal neovascular membranes using OCTA in patients with treatment-naïve, continuously treated, and previously treated exudative AMD. Their results showed that the most frequently identified membrane morphology was well-defined (medusa or sea-fan), which occurred in 69% of eyes with NE-MNV and in 77% eyes receiving ongoing anti-VEGF treatment. Conversely, long-filamentous morphology was the most frequent type in the previously treated group (53%) [8].

Using the OCTA technique to observe pachychoroid neovasculopathy (PNV), significant differences in the morphology of exudative and non-exudative PNV were identified. The results showed that the presence of an indistinct network of neovascular vessels or long, thread-like linear vessels is more commonly associated with non-exudative PNV. According to the study, lacy wheel and the sea fan patterns were observed in 86.1% of eyes in the exudative PNV and 33.3% in the nonexudative PNV group. Anastomosis or loops were identified in 94.4% of the exudative group, versus 46.6% in the non-exudative PNV group [14].

It seems that there are specific differences between non-exudative MNV secondary to AMD and pachychoroid spectrum diseases.

These differences have been demonstrated in studies using OCT and en face infrared reflectance (IR) technology. The results showed that non-exudative MNVs in pachychoroid eyes are characterized by sharper and more inhomogeneous pigment epithelium detachments and lighter choroidal shadowing compared to NE-MNVs in AMD eyes. Furthermore, they often display a hyporeflective halo around the lesion when evaluated with IR imaging [15]. In our study, we also assessed the condition of the fellow eye, which was normal in 40.63% of cases. Dry AMD was the most commonly observed pathology in the second eye. Interesting results were presented in a study assessing the occurrence of subclinical neovascularization without exudation in the second eye in patients with unilateral typical exudative AMD or polypoidal choroidal vasculopathy (PCV) using indocyanine green angiography (ICGA) and OCTA. While the prevalence of non-exudative neovascularization in the second eye was 18%, pachychoroid pigment epitheliopathy was the only risk factor associated with non-exudative neovascularization [16]. In our observations, PPE was the etiology of NE-MNV in 12 of the studied 32 eyes (37.5%). Furthermore, it occurred in two fellow eyes (6.25%), a prevalence similar to that of central serous choroidopathy (CSC). A significant feature to note is the presence of the DLS and the TLS. Both of them are identified on OCT scans and may indicate the presence of NE-MNV. In our study, the DLS and/or the TLS were one of the inclusion criteria due to the correlation between this feature and a high probability of developing MNV. Other authors have also emphasized the important role played by the presence of these signs. The DLS refers to a shallow separation between the retinal pigment epithelium and Bruch’s membrane, whereas the TLS is a distinct finding that involves the RPE, neovascular tissue, and Bruch’s membrane. Capuano et al. assessed the sensitivity and specificity of the TLS on structural OCT images for the diagnosis of treatment-naïve non-exudative type-1 MNV in AMD. The results of the study demonstrated high sensitivity and specificity of this feature in diagnosing treatment-naïve Type 1 non-exudative MNV [17]. However, it should be noted that the presence of DLS/TLS does not always confirm the presence of concurrent MNV. In our cohort, 32 of 51 eyes (62.7%) presenting with the DLS and/or TLS were found to be associated NE-MNV.

Whereas most studies on NE-MNV focus on its occurrence in the course of AMD, in our report, 40.62% of cases were associated with other pathologies, mainly pachychoroid pigment epitheliopathy. Therefore, we should also be aware of this type of pathology.

Pachychoroid spectrum disease is a group of disorders characterized by a thickened choroid, dilated outer choroidal vessels (pachyvessels), and thinning of the overlying inner choroid. The spectrum includes conditions like central serous chorioretinopathy, pachychoroid pigment epitheliopathy, pachychoroid neovasculopathy, polypoidal choroidal vasculopathy, and peripapillary pachychoroid syndrome. As is well known, one of the complications of pachychoroid disease may be the development of pachychoroid neovasculopathy, which is a distinct form of type 1 MNV.

Other research also indicates the presence of other relevant prognostic biomarkers for NE-MNV. They include: the rate of growth and perfusion density of the MNV [1], vessel density (VD) of the superficial vascular plexus (SVP) and deep vascular complex (DVC) [18], the presence of a shallow irregular retinal pigment epithelial elevation (SIRE) [11], and the size of the lesion [10].

Current clinical recommendations for eyes with NE-MNV emphasize strict monitoring and frequent follow-up visits to ensure prompt anti-VEGF therapy upon conversion [6,19].

Scientific reports demonstrate that in patients with untreated non-exudative MNV lesions secondary to AMD, the disease may remain stable and asymptomatic for extended periods. Furthermore, this non-eudative state may even be beneficial in terms of protection against atrophy [2,5,20]. Thus, aggressive treatment of such lesions is not recommended.

It is crucial to identify eyes at high risk of developing exudation in the near future. This is particularly important because NE-MNV is an asymptomatic condition.

We know that earlier treatment of exudation leads to better visual acuity outcomes, yet we still lack a robust clinical strategy for predicting progression. Therefore, the current management approach involves identifying high-risk eyes through frequent follow-up visits, routine OCT and OCTA examinations, and empowering patients to recognize symptoms associated with conversion [21]. Further prospective studies involving larger groups of patients are necessary to more accurately assess the clinical features of MNV and determine appropriate management strategies.

### Limitations and Strengths

Our study has certain limitations, including a relatively small patient cohort, variable observation periods, and its retrospective design. Moreover, the use of a 9 mm radial OCT B-scan centered on the fovea may fail to detect focal exudation between scan lines. However, a major strength of this study is the inclusion and observation not only of AMD patients but also of the PPE subgroup.

## Figures and Tables

**Table 1:** Baseline characteristics of the study group.

Analyzed Categorical Traits	*n* (%)
**No. of participants**	30
**No. of eyes**	32
**Gender:**	
▪ **Female**	15 (50.00)
▪ **Male**	15 (50.00)
**Cause of NE-MNV:**	
• **AMD**	19 (59.38)
• **Non-AMD**	13 (40.62)
○ **Pachychoroid**	12 (37.50)
○ **Others**	1 (3.12)
**Double and/or triple-layer sign:**	
• **Present**	32 (100.00)
• **Absent**	0 (0.00)
**Fluid:**	
• **Absent**	32 (100.00)
**Morphology of the MNV:**	
• **Glomerulus**	7 (21.88)
• **Indistinct**	8 (25.00)
• **Long linear vessels**	7 (21.88)
• **Medusa**	9 (28.12)
• **Seafan**	1 (3.12)
**Conditions in the fellow eye:**	
• **Abnormal:**	19 (59.38)
○ **Dry AMD**	6 (18.75)
○ **Exudative AMD**	3 (9.37)
○ **CSC**	2 (6.25)
○ **NE-MNV**	4 (12.50)
○ **Epitheliopathy**	2 (6.25)
○ **Pachychoroid**	2 (6.25))
• **Normal**	13 (40.62)
**Analyzed numerical traits**	**M (SD), Me (Q_1_–Q_3_)**
**Age [y]**	64.09 (15.48), 66 (50–66)
**Choroidal thickness [µm]**	293.45 (136.99), 304 (171–365)
**Central macular thickness [µm]**	223.93 (40.02), 223 (206–253)

(Abbreviations: *n*—number, %—percentage, M—mean, SD—standard deviation, Me—median, Q—quartiles; NE-MNV—non-exudative macular neovascularization, AMD—age-related macular degeneration, CSC—central serous chorioretinopathy).

**Table 2:** Presence of exudation—subretinal fluid in the study eye by selected variables.

Categorical Traits	Fluid Present (*n* = 5)	Fluid Absent (*n* = 27)	*p* Value
	*n* (%)	*n* (%)	
**Gender:**			
▪ **Female**	2 (40.00)	14 (51.85)	>0.9999
▪ **Male**	3 (60.00)	13 (48.15)	
**Cause of NE-MNV:**			
• **AMD**	4 (80.00)	15 (55.56)	0.6247
• **Non-AMD**	1 (20.00)	12 (44.44)	
**Morphology:**			
• **Glomerulus**	0 (0.00)	7 (25.93)	0.2087
• **Indistinct**	0 (0.00)	8 (29.63)	
• **Long linear vessels**	2 (40.00)	5 (18.52)	
• **Medusa**	3 (60.00)	6 (22.22)	
• **Sea fan**	0 (0.00)	1 (3.70)	
**Fellow eye:**			
• **Abnormal:**	4 (80.00)	15 (55.56)	0.6247
• **Normal**	1 (20.00)	12 (44.44)	
**Numerical traits**	**M (SD), Me (Q_1_–Q_3_)**	**M (SD), Me (Q_1_–Q_3_)**	
**Age [y]**	62.12 (7.85), 64 (56–67)	64.45 (16.59), 71 (48–79)	0.6792
**Choroidal thickness [µm]**	360.40 (153.83), 452 (311–455)	279.50 (132.49), 279 (169–361)	0.0938
**Central macular thickness [µm]**	199.80 (52.82), 182 (160–231)	228.96 (36.22), 225 (208–256)	0.0938

(Abbreviations: *n*—number, %—percentage, M—mean, SD—standard deviation, Me—median, Q—quartiles; NE-MNV—non-exudative macular neovascularization, AMD—age-related macular degeneration, CSC—central serous chorioretinopathy. Details regarding statistical significance testing: the data in the subsequent cross-tables were verified using Fisher’s exact test; the Mann–Whitney U test was used for testing the between-group difference in the subjects’ age; all *p*-values are given for exact calculations).

## Data Availability

The data presented in this study are available upon request from the corresponding author due to privacy.
